# Correction: Li et al. African Swine Fever Virus: A Review. *Life* 2022, *12*, 1255

**DOI:** 10.3390/life14030381

**Published:** 2024-03-14

**Authors:** Zhaoyao Li, Wenxian Chen, Zilong Qiu, Yuwan Li, Jindai Fan, Keke Wu, Xiaowen Li, Mingqiu Zhao, Hongxing Ding, Shuangqi Fan, Jinding Chen

**Affiliations:** 1 College of Veterinary Medicine, South China Agricultural University, Guangzhou 510642, China; lzhaoyao123@163.com (Z.L.); chwenxian0912@163.com (W.C.); qiuzilong003@163.com (Z.Q.); lyw13253374768@163.com (Y.L.); fanjindai@stu.scau.edu.cn (J.F.); 13660662837@163.com (K.W.); 18306616234@163.com (X.L.); zmingqiu@scau.edu.cn (M.Z.); dinghx@scau.edu.cn (H.D.); 2 Guangdong Laboratory for Lingnan Modern Agriculture, Guangzhou 510642, China; 3 Key Laboratory of Zoonosis Prevention and Control of Guangdong Province, Guangzhou 510642, China

## Figure Legend

In the original publication [1], the legends in Figures 2 and 4 of the review do not clearly indicate the source of the picture. Add “(adapted with permission from Alejo et al. [49])” to the end of the legend in Figure 2. Add “(adapted with permission from Andres and Salas [47])” to the end of the legend in Figure 4. The authors state that the scientific conclusions are unaffected. This correction was approved by the Academic Editor. The original publication has also been updated.
